# Low treatment completion rates reveal gaps in the LTBI care cascade among university students

**DOI:** 10.3389/ftubr.2025.1710215

**Published:** 2025-11-06

**Authors:** Anu K. Murthy, Richard A. Goodman, Miranda A. Moore

**Affiliations:** 1 Department of Family and Preventive Medicine, Emory University School of Medicine, Atlanta, GA, United States; 2 Department of Internal Medicine, Emory University School of Medicine, Atlanta, GA, United States

**Keywords:** latent tuberculosis infection, international students, treatment adherence, cascade of care, student health, healthcare students

## Abstract

**Background:**

University students, particularly those from tuberculosis (TB)-endemic countries and students in health professions, are at increased risk for latent tuberculosis infection (LTBI). In the United States (U.S.), TB screening is required for applicants for permanent residency and refugee status but not for individuals entering on student visas, and institutional policies vary. At our university, which requires pre-matriculation TB screening for all incoming students, low LTBI treatment completion rates prompted a review of care delivery.

**Methods:**

We conducted a retrospective chart review of students diagnosed with LTBI at a medium-sized private university from 2018 to 2023. Records identified by ICD-10 codes were reviewed for demographics, school enrollment, testing method, regimen, and treatment outcome.

**Results:**

Of 687 students with TB-related visits, 82 (12%) were diagnosed with LTBI. Median age was 27 years (interquartile range 23–31); 55% were female and 65% were non-U.S.-born, most often from China and India. Students represented nine schools, with Medicine (17%) and Nursing (14%) comprising about one-third of cases. Forty-eight students (59%) initiated treatment, while 34 (41%) did not, primarily due to declining therapy or incomplete follow-up. Among those treated, 22 (46%) completed therapy, corresponding to 27% overall. Completion was highest for nine months of isoniazid (88%) and lowest for 4 months of rifampin (17%). Several documentation and follow-up gaps were identified.

**Conclusion:**

Losses across the LTBI care cascade were common in this university setting and reflect patterns reported in other U.S. and international studies. Strengthening education, follow-up, access to shorter regimens, and documentation may improve completion rates and support TB elimination goals.

## 1 Introduction

Globally, an estimated one-quarter of the population is infected with *Mycobacterium tuberculosis*, and management of latent tuberculosis infection (LTBI) is central to elimination strategies (1, 2). The WHO End TB Strategy highlights LTBI testing and treatment among high-risk groups—including healthcare personnel and internationally mobile populations—as essential to reducing TB incidence (3). In the United States, LTBI is not consistently reported across states, leading the Centers for Disease Control and Prevention (CDC) to rely on survey-based estimates and modeling (4, 5). Prevalence is estimated at 8.6 million people (95% CI: 8.3–8.8 million) (6). While only 5%−10% of untreated LTBI progresses to active TB, nearly 80% of reported TB arises from this pool, and non-U.S.-born persons account for most cases (7).

Comparisons across countries highlight the role of migration and mobility. Between 2000 and 2011, TB incidence declined in the United States but increased in the United Kingdom, with foreign-born populations driving both patterns (8). In the U.S., surveillance now shows that case counts and incidence rose during 2021–2023, exceeding pre-COVID levels (9). These trends highlight the importance of strengthening LTBI detection and treatment.

University students, particularly those studying abroad, represent a globally mobile population that may face unique challenges in LTBI testing, treatment initiation, and follow-up. Studies show higher LTBI prevalence in mobile populations and substantial variation in treatment completion among migrants (10, 11). U.S. college-age analyses further demonstrate markedly higher LTBI prevalence in foreign-born young adults than U.S.-born peers (12). Screening policies across U.S. colleges vary widely; a national survey found that only two-thirds of institutions had TB screening policies, typically targeting international students or selected programs (13). Our university health service follows American College Health Association (ACHA) guidelines for targeted TB screening (14). This study aimed to describe LTBI demographics, treatment regimens, and completion in a medium-sized private U.S. university, with emphasis on international and healthcare students.

## 2 Methods

### 2.1 Study design

**Figure 1:**
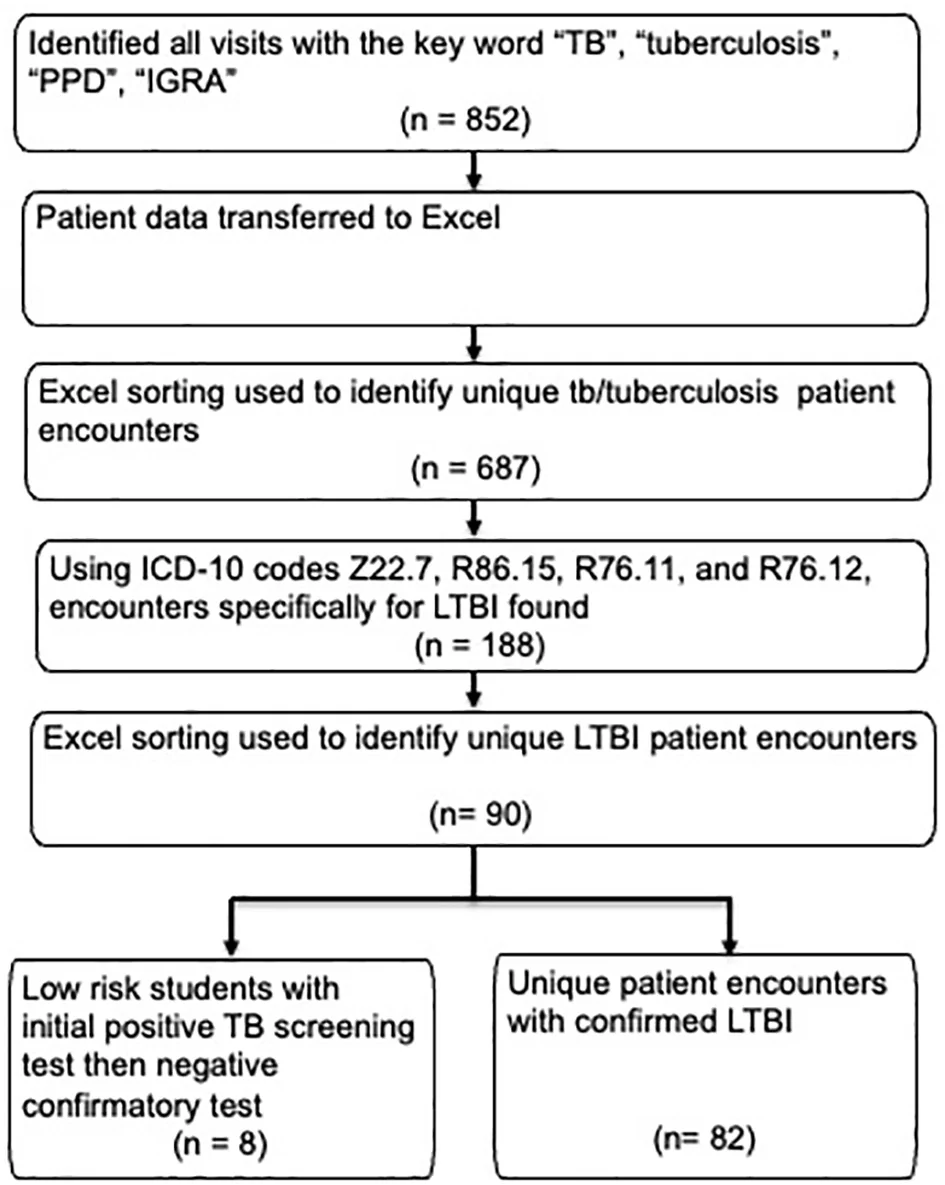
Data sorting and inclusion flowchart for LTBI cases. LTBI, latent tuberculosis infection; TB, tuberculosis; PPD, purified protein derivative; IGRA, interferon Gamma Release Assay.

This study was a retrospective analysis of electronic medical records to evaluate latent tuberculosis infection (LTBI) screening, diagnosis, and treatment among incoming university students. Electronic medical records were searched using “TB,” “tuberculosis,” “PPD,” “IGRA,” and ICD-10 codes (Z22.7, R76.11, R76.12, and Z86.15). Extracted data included demographics, school enrollment, diagnostic test type, chest radiograph results, treatment regimen, and completion status. Completion was defined as documentation of regimen completion. De-identified data were exported into Excel for descriptive analysis using pivot tables. Electronic medical record notes were manually reviewed to confirm LTBI diagnosis, prescribed regimen, reasons for treatment refusal or discontinuation, and completion status. See Figure 1 for details on data sorting.

### 2.2 Study population

The study included incoming students at a medium-sized private university in Atlanta, Georgia, with approximately 16,000 students, including around 4,300 international students from more than 100 countries, primarily China and India. Students were included if they underwent TB screening per university protocols. No students in this study were diagnosed with active TB, reported known exposure to active TB, exhibited symptoms of active TB, or had HIV or other criteria indicating high risk for disease progression.

### 2.3 Intervention and exposures

All incoming students completed a TB risk questionnaire based on ACHA guidelines (14). Students screening positive underwent targeted testing with either tuberculin skin tests (TST) or Interferon-Gamma Release Assays (IGRAs; QuantiFERON-TB Gold Plus or T-SPOT.TB). IGRAs were preferred for students with prior bacille Calmette-Guérin (BCG) vaccination. Students unable to complete testing before arrival were tested on campus or referred to the local health department if on-campus testing was cost-prohibitive. Confirmatory testing with a second assay was performed at provider discretion for borderline or questionable false results. All students with a positive test underwent chest radiography to exclude active TB.

LTBI treatment followed recommendations from the National Tuberculosis Controllers Association and CDC (15), which endorse four regimens: 3HP (isoniazid + rifapentine, weekly × 3 months), 4R (rifampin, daily × 4 months), 3HR (isoniazid + rifampin, daily × 3 months), and 9H (isoniazid, daily × 9 months). Regimen choice was made jointly by providers and students. After 2019, the clinic discontinued stocking rifapentine, halting in clinic 3HP and its directly observed therapy (DOT) protocol; 3HP was switched to self-administered therapy (SAT) and sourced from local pharmacies, while isoniazid (INH) became the only regimen dispensed on-site. Rifamycins were otherwise obtained from local pharmacies.

### 2.4 Outcomes

The primary outcomes were LTBI diagnosis, treatment regimen selection, and treatment completion status. LTBI was defined as a positive TB test [TST or interferon Gamma Release Assay (IGRA)] with a negative chest radiograph for active TB. Completion was defined as documentation of regimen completion in the electronic medical record. Data on demographics, school enrollment, diagnostic test type, chest radiograph results, prescribed regimen, reasons for treatment refusal or discontinuation, and completion status were extracted and analyzed.

### 2.5 Ethical approval

The Emory Institutional Review Board deemed this project to not meet the definition of human subject research, under the category of clinical quality improvement.

## 3 Results

From 2018 to 2023, the student health clinic recorded 852 encounters related to TB concerns, involving 687 unique students. Eighty-two of 687 students (12%) with TB-related visits were diagnosed with untreated LTBI. Eight students out of the 687 were identified to have previously treated LTBI. Median age was 27 years (IQR 23–31), 55% were female, and 65% were non-U.S.-born, most frequently from China (12%) and India (11%). Students represented nine schools, including Medicine (17%) and Nursing (14%). See Table 1 for the summary of student characteristics data.

**Table 1:** Characteristics of university students diagnosed with LTBI, 2018–2023 (*n* = 82).

**Characteristic**	***n* (%)**
**Demographics**	
Age, years, median (IQR, range)	27 (23–31, 17–70)
Female	45 (55)
Male	37 (45)
**Birth country**	
U.S.-born	29 (35)
Non-U.S.-born	53 (65)
China	10 (12)
India	9 (11)
Rwanda	4 (5)
Vietnam	3 (4)
Bangladesh	3 (4)
Nigeria	3 (4)
Afghanistan	2 (2)
Other countries (*n* = 19, one each)^a^	19 (23)
**School enrollment**	
Graduate arts and sciences	15 (18)
Medicine	14 (17)
Undergraduate arts and sciences	13 (16)
Graduate business	12 (15)
Nursing (graduate + undergraduate)	12 (14)
Law	7 (9)
Public health	6 (7)
Undergraduate business and theology^a^	3 (3)
**LTBI testing**	
One test per student	62 (76)
Two tests per student	20 (24)
**LTBI treatment status**	
Treatment prescribed	48 (59)
Treatment not prescribed	34 (41)
**Reasons for not prescribing treatment**	
Declined	12 (15)
Lost to follow-up after initial interest	9 (11)
Referred externally (health department/ID specialist)	7 (9)
Treated by outside provider	6 (7)

^a^Countries with one student are not listed to protect privacy. Schools with less than three students are grouped together to protect privacy.

LTBI, latent tuberculosis infection; IQR, interquartile range; U.S., United States; ID, infectious disease.

**Figure 2:**
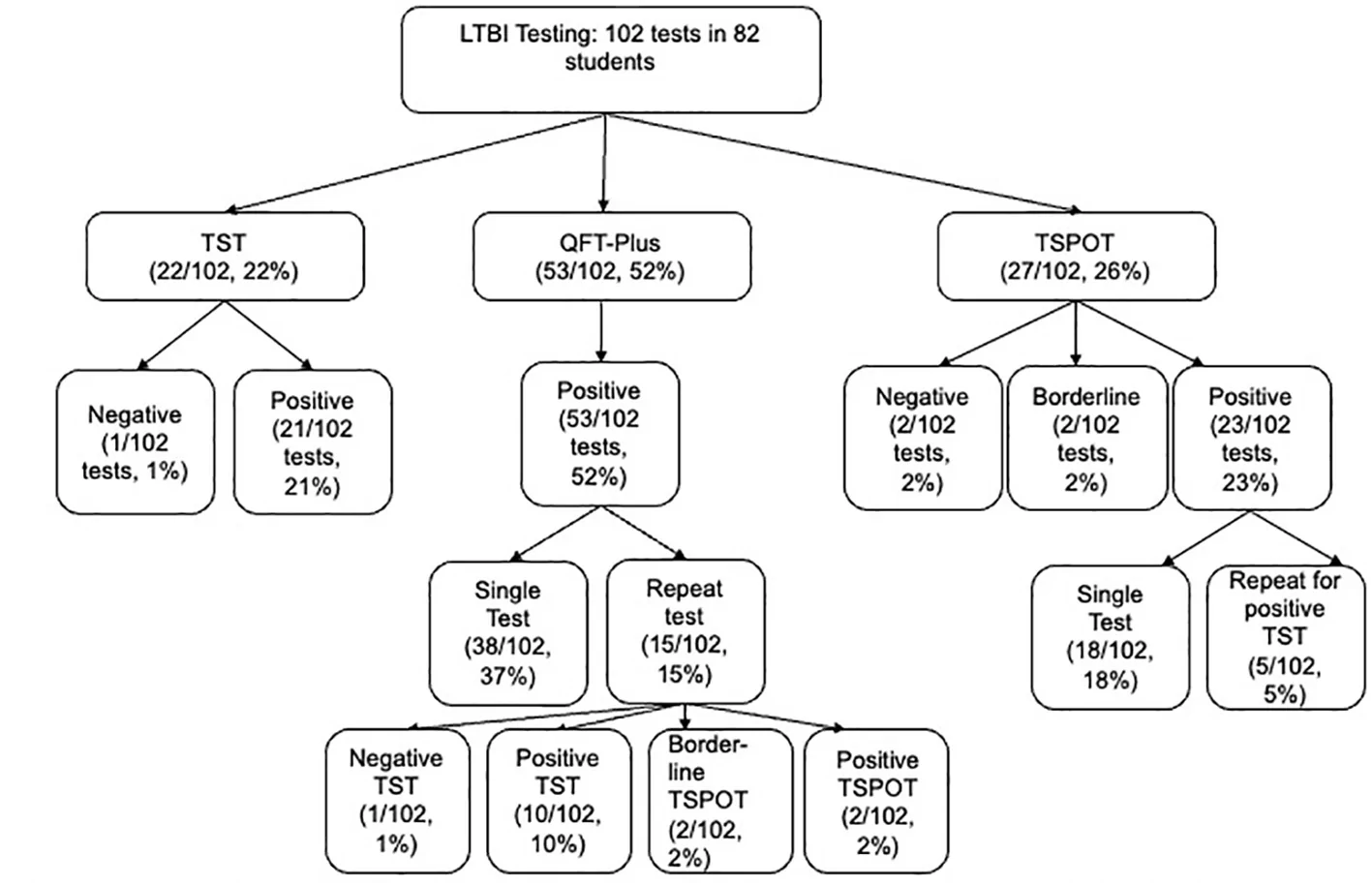
LTBI testing flow among screened students. LTBI, latent tuberculosis infection; TST, tuberculin skin test; QFT-Plus, QuantiFERON-TB Gold Plus; TSPOT, T-SPOT.TB. Distribution of 102 tests (TST 22%, QTF-Plus 52%, TSPOT 26%) among 82 students, including positive test (95%), negative test (3%), borderline (2%). Single test (81%) for 60 students and repeat test (20%) for 20 students.

A total of 102 screening tests were performed: 22 TST (22%), 53 QuantiFERON (52%), and 27 T-SPOT (26%). Twenty students (24%) underwent confirmatory testing, most often IGRA after a positive TST. See Figure 2 for the testing flow.

Of the 82 untreated LTBI students, 48 (59%) initiated therapy and 34 (41%) did not. Reasons for non-initiation among the 34 students included declining therapy 12 (35%), incomplete follow-up after initial interest 9 (26%), referral to external providers (health department or infectious disease) 7 (21%), or outside treatment 6 (18%).

Among students treated at our clinic, 18 (38%) received 3HP, 12 (25%) 4R, 10 (21%) 3HR, and 8 (17%) 9H. Documented completion was 58% (28/48). Completion was highest for 9H (88%), followed by 3HP (50%), 3HR (40%), and 4R (17%). Within 3HP, completion was higher with directly observed therapy (67%) than self-administered therapy (42%).

**Figure 3:**
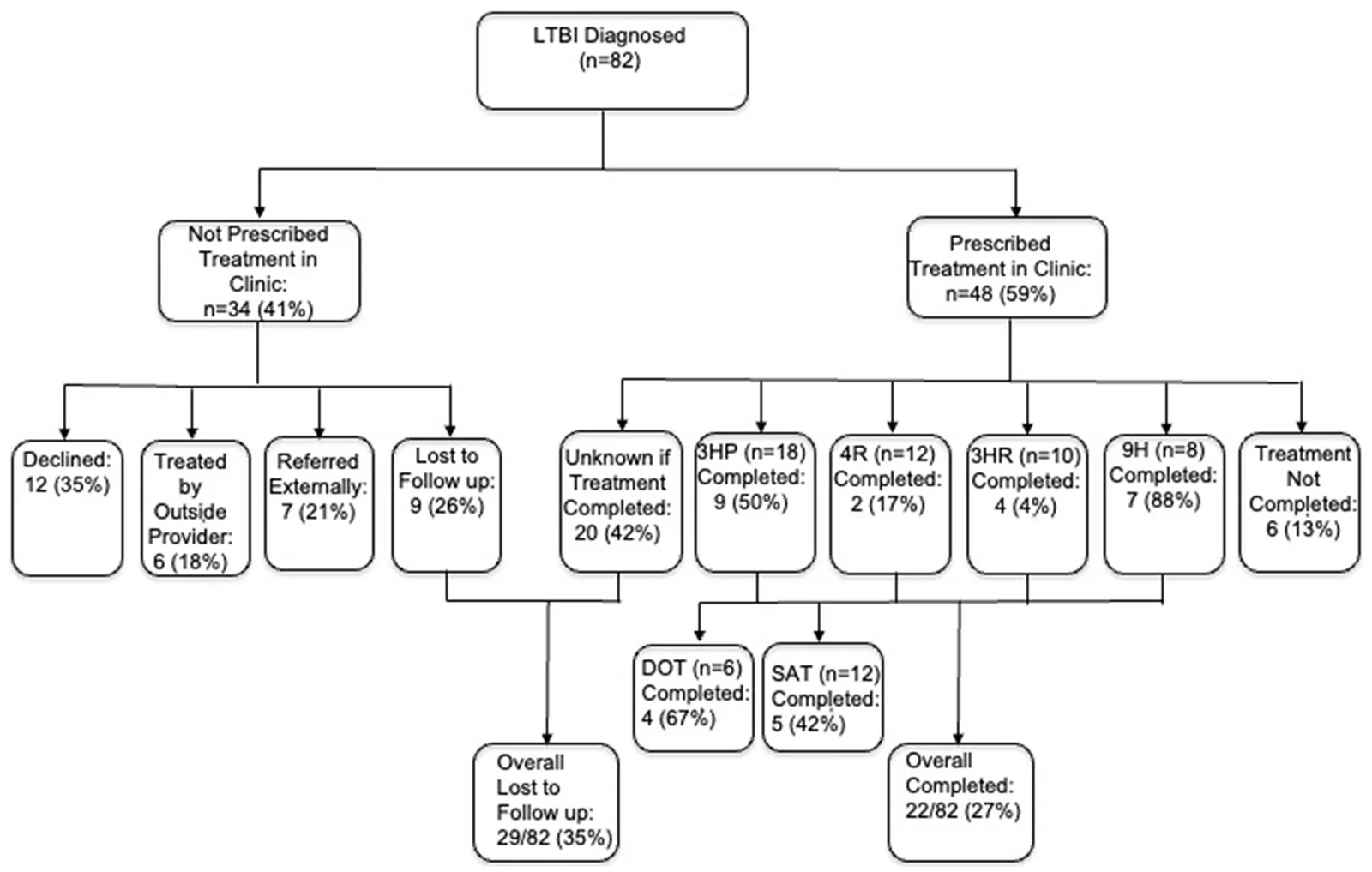
Cascade of care from LTBI screening to treatment completion. LTBI, latent tuberculosis infection; 3HP, 3 months isoniazid + rifapentine; 4R, 4 months rifampin; 3HR, 3 months isoniazid + rifampin; 9H, 9 months isoniazid. Of 82 diagnosed students, 59% clinic prescribed treatment (3HP, 4R, 3HR, 9H regimens); 41% not prescribed; overall completion 27%, overall lost to follow-up 35%.

The full cascade from screening to treatment completion is shown in Figure 3.

## 4 Discussion

This study describes the full LTBI cascade of care within a U.S. university health setting, with specific attention to international and healthcare students—two groups consistently identified as higher risk but rarely examined in this context.

Our study shows substantial gaps in latent tuberculosis infection (LTBI) care among university students. Only 59% of students diagnosed with LTBI began treatment, and fewer than two-thirds of those completed therapy, leaving an overall completion rate of 27%.

These findings are consistent with broader experience. Alsdurf et al. reported that fewer than one in five people worldwide complete LTBI treatment after diagnosis (16). Our figures-−59% initiation and 58% completion among those who started—suggest that university students face many of the same barriers.

International students accounted for nearly two-thirds of LTBI cases at our university. Collins et al. found that international students in the U.S. have a significantly higher TB case rate than the general foreign-born population (17). Healthcare students represented about one-third of cases and are also a priority group in global TB strategies (18). These observations highlight the need for interventions tailored to university settings.

Regimen-specific outcomes offer further insights. Unexpectedly, the 9H regimen had the highest completion rate in our cohort, likely due to its lower cost, on-site dispensing at the student health clinic, and structured adherence support. Students on 9H received monthly prescriptions without additional copays, with costs billed directly to insurance. By contrast, programmatic studies in U.S. public health clinics have reported completion rates above 80% with 3HP, even among persons experiencing homelessness or incarceration (19). In a Seattle cohort, McClintock and colleagues found 85% completion with both 3HP and 4R, compared with just 52% for 9H, independent of monitoring type (20). These data reinforce that regimen choice is a strong predictor of adherence, although barriers specific to university students—academic schedules, insurance coverage, and limited follow-up—may override those advantages.

In our study, 3HP completion fell from 67% with directly observed therapy (DOT) to 42% with self-administered therapy (SAT) during the COVID-19 pandemic. This pattern is consistent with the PREVENT TB and iAdhere trials, which showed higher adherence with DOT than SAT (21, 22). A systematic review also noted wide variation in adherence to rifapentine-based regimens (23). Together, these results show that regimen choice alone is not enough—cost, side effects, monitoring, and access are equally important.

National data point in the same direction. Insurance claims analyses show that fewer than half of patients complete isoniazid monotherapy, and some progress to active TB despite documented completion (24). Similarly, commercially insured cohorts show completion rates below 50% for isoniazid, comparable to those seen in public health programs and in our university population (25). Qualitative work in an integrated health system found that patients often reported limited knowledge about LTBI, side effects, lack of support, and doubts about the value of treatment as reasons for not starting or completing therapy (26). Similar concerns have been noted elsewhere; a survey of more than 15,000 college students in Wuhan, China found gaps in TB knowledge and inconsistent prevention behaviors, highlighting the need for student-focused education (27).

Our results align with current U.S. guidance. Eastment et al. emphasized that education, access to care, and active monitoring are key to treatment completion in high-risk groups (28). The 2020 NTCA/CDC guidelines recommend shorter rifamycin-based regimens to improve adherence (15), and the 2023 U.S. Preventive Services Task Force advises routine LTBI screening for high-risk adults, including non-U.S.-born persons common in universities (29). These recommendations underscore the role of university health services in TB prevention.

Among the 34 students who did not begin treatment at our clinic, 12 declined for unspecified reasons, nine were lost to follow-up, six chose to see an outside provider, and seven were referred externally to the health department or infectious disease specialist. Limited documentation prevented us from determining reasons for non-initiation or confirming treatment completion for the 13 students managed off campus. This points to the need for better record-keeping, systematic follow-up, and outreach to address student concerns.

### 4.1 Implications

Emory already requires universal pre-matriculation TB screening and targeted testing for students flagged as high risk via the screening questionnaire. Despite these measures, treatment initiation and completion remain suboptimal. The following low-resource, actionable items could strengthen the LTBI cascade:

1. Pre-departure option for admitted students from high-incidence countries. Offer an overseas pre-arrival pathway that includes IGRA testing, prompt clinical evaluation to exclude active disease, and access to short-course preventive therapy (e.g., 3HP or 4R), with remote support for follow-up and adherence. Evidence from U.S.-bound immigrants shows high completion with pre-departure 3HP (30).
2. Rapid start on arrival. For non-U.S.-born students and those entering health-professional programs, implement a fast-track process on campus: same-day evaluation after a positive test, immediate offer of short-course therapy, and staff help with scheduling and medication pick-up.
3. Strong recommendation with documented declination. Rather than a mandate, strongly recommend LTBI treatment and document informed declination for those who refuse, with continued symptom review. Include a statement that students may reconsider treatment at any time.
4. Reduce practical barriers. Provide medications at no or low cost; streamline laboratory monitoring when indicated; use multilingual education materials; send simple reminders (secure message/text); and, when appropriate, use video directly observed therapy to support adherence.
5. Use EMR systems for treatment tracking and support. Standardized EMR templates and dashboards can track each step of the cascade (screened → tested → evaluated → initiated → completed), trigger recalls for missed steps, and generate routine reports on time to treatment, regimen selection, completion, and adverse events. Because LTBI reporting requirements vary by state, campus-level EMR tracking is essential for visibility and accountability.

In the current U.S. environment—where LTBI reporting and treatment requirements are variable—these targeted, low-resource actions are feasible next steps that can meaningfully improve initiation and completion and reduce the risk of TB reactivation in university settings.

## 5 Strengths and limitations

This study has limitations. It was conducted at a single institution, limiting generalizability. Small regimen groups reduced the precision of completion estimates. After 2019, the student health clinic discontinued stocking 3HP and ceased DOT, stocking only INH with SAT for all treatment options. This shift likely influenced regimen selection and completion rates, as shorter regimens like 3HP have higher adherence in other settings (19, 20). Conversely, the policy of dispensing INH in monthly increments—provided on-site without additional copays—may have boosted adherence for INH compared to regimens requiring off-site management. The retrospective design restricted information on reasons for non-initiation or incomplete therapy. In addition, screening tests (TST, QuantiFERON Gold Plus, T-SPOT.TB) have inherent diagnostic limitations. Despite these issues, our findings point to practical steps—especially stronger documentation and targeted support for high-risk group—that other clinics can adopt to improve LTBI care.

## 6 Conclusion

University students with LTBI encounter substantial barriers to initiating and completing treatment, consistent with global patterns yet exacerbated by demanding academic schedules, restricted healthcare access, and tuberculosis-related stigma. Enhanced follow-up, access to shorter regimens, improved documentation, and targeted support for international and healthcare students can enable university health services to address these gaps and advance TB prevention.

## Data Availability

The raw data supporting the conclusions of this article will be made available by the authors, without undue reservation.
